# In Vitro Evaluation of ACE and DPP-IV Inhibitory, and GLP-1 Stimulation Activities of Collagen Hydrolysate Enriched in Tripeptides

**DOI:** 10.3390/biomedicines14030589

**Published:** 2026-03-05

**Authors:** Melissa Fanzaga, Lorenza d’Adduzio, Carlotta Bollati, Maria Silvia Musco, Giovanna Boschin, Gilda Aiello, Carmen Lammi

**Affiliations:** 1 Department of Pharmaceutical Sciences, University of Milan, 20133 Milan, Italy; 2 Department of Human Science and Quality of Life Promotion, Telematic University San Raffaele, 00166 Rome, Italy

**Keywords:** ACE, collagen peptides, Caco-2 cells, DPP-IV, GLP-1

## Abstract

**Background/Objectives:** Collagen hydrolysates are widely used as nutritional ingredients for skin and joint health; however, growing evidence indicates that collagen may also exert beneficial effects on cardiometabolic pathways. Short peptides have been shown to modulate angiotensin-converting enzyme (ACE) and dipeptidyl peptidase IV (DPP-IV), key regulators of blood pressure and glucose homeostasis. This study aimed to assess the dual ACE- and DPP-IV inhibitory and GLP-1 stimulation activities, respectively of a tripeptide-enriched formulation (CH). The study was performed using a benchmark collagen hydrolysate (BCH) as reference. **Methods:** ACE and DPP-IV inhibitory activities were evaluated using in vitro enzymatic assays. Cellular compatibility and in situ DPP-IV inhibition were assessed in Caco-2 intestinal cells, while glucagon-like peptide-1 (GLP-1) secretion was measured in STC-1 enteroendocrine cells. The degree of hydrolysis was determined by OPA assay, and nanoLC–HRMS was used to characterize and compare the proteomic profiles of the samples. **Results:** Both hydrolysates exhibited dose-dependent ACE and DPP-IV inhibition; however, CH showed significantly higher inhibitory activity at comparable concentrations. CH also reduced cellular DPP-IV activity in Caco-2 cells and stimulated GLP-1 secretion in STC-1 cells, whereas BCH showed limited or non-significant cellular effects. Peptidomic analysis revealed an enrichment of short- and medium-length peptides in CH, while BCH contained a higher proportion of long peptides (>2000 Da). Consistently, CH exhibited a 1.7-fold higher degree of hydrolysis than BCH. **Conclusions:** The tripeptide-enriched collagen hydrolysate demonstrated superior enzymatic and cellular bioactivity compared with the benchmark formulation, supporting its potential as a multifunctional bioactive ingredient for health applications.

## 1. Introduction

Collagen is one of the most abundant structural proteins in mammals and has been extensively investigated as a dietary ingredient, primarily in relation to skin health, joint function, and connective tissue integrity. In this context, collagen hydrolysates are widely used in nutritional interventions, and multiple clinical studies have reported improvements in skin elasticity, wrinkle depth, and dermal matrix organization following oral supplementation, typically at daily doses ranging from 2.5 to 5 g [1]. These effects have been largely attributed to the high bioavailability of low-molecular-weight collagen peptides and their role in supporting extracellular matrix turnover and dermal fibroblast activity.

In parallel with these established applications, increasing attention has been directed toward the potential interaction of food-derived collagen peptides with metabolic pathways relevant to cardiovascular and glucose homeostasis, thereby motivating investigation beyond the traditional skin–joint axis.

Cardiometabolic disorders, including hypertension and type 2 diabetes mellitus (T2DM), represent major global health challenges, with a continuously increasing prevalence and a substantial socioeconomic burden. These conditions frequently coexist and share overlapping pathophysiological mechanisms, such as dysregulation of the renin–angiotensin system (RAS), endothelial dysfunction, chronic low-grade inflammation, and impaired incretin signaling [2]. Consequently, nutritional strategies targeting multiple metabolic pathways are increasingly investigated as complementary approaches for cardiometabolic risk management. Among the molecular targets involved in blood pressure regulation, angiotensin-converting enzyme (ACE) plays a central role by catalyzing the conversion of angiotensin I into the potent vasoconstrictor angiotensin II and by promoting the degradation of vasodilatory peptides such as bradykinin. Inhibition of ACE remains a cornerstone of antihypertensive pharmacotherapy; however, growing attention has been directed toward food-derived bioactive peptides as natural ACE inhibitors with potential preventive and adjunctive therapeutic value [3]. In this context, collagen-derived peptides have emerged as particularly promising candidates, due to their structural features, abundance of proline- and hydroxyproline-containing motifs, and documented ACE-inhibitory activity in vitro and in vivo [4]. Concomitantly, glycemic control is critically modulated by incretin hormones, particularly glucagon-like peptide-1 (GLP-1), which enhances glucose-dependent insulin secretion, suppresses glucagon release, and contributes to appetite regulation. The biological activity of GLP-1 is limited by its rapid degradation mediated by dipeptidyl peptidase IV (DPP-IV), making DPP-IV inhibition a validated therapeutic strategy for T2DM management [5,6]. Beyond pharmaceutical agents, several studies have demonstrated that dietary peptides can exert DPP-IV inhibitory activity and, in some cases, stimulate GLP-1 secretion, thereby supporting glucose homeostasis through nutritionally relevant mechanisms [7]. Collagen represents a unique protein source for the generation of bioactive peptides and increasing evidence suggests that short collagen-derived peptides may exert multifunctional biological effects, including ACE inhibition, modulation of endothelial function, and regulation of incretin-related pathways [8]. Comparative evaluation of distinct collagen hydrolysates generated through different enzymatic strategies represents an underexplored area, despite its relevance for the rational design of peptide-based functional ingredients. However, despite the growing body of evidence on collagen-derived peptides, most available studies have focused on isolated enzymatic assays or single biological endpoints, while integrated investigations combining enzymatic, cellular, and proteomic approaches remain limited.

In this framework, the present study aimed to evaluate the ACE and DPP-IV inhibitory and GLP1 stimulation activity potential of CollaSel TRIPEPTIDE (CH), using another commercially available collagen hydrolysate (BCH) as benchmark. A multidisciplinary experimental approach was employed, integrating in vitro enzymatic assays for ACE and DPP-IV inhibition, intestinal cellular models (Caco-2 and STC-1 cells) to assess cellular compatibility, enzyme inhibition in situ, and GLP-1 secretion, as well as high-resolution peptidomic profiling to characterize their compositions.

Actually, CH is characterized by a higher degree of hydrolysis compared to the benchmark product (BCH). Importantly, while CH is more extensively hydrolyzed and enriched in tripeptides, the benchmark product is not declared to be enriched in tripeptides. The primary objective of this study was to determine whether a greater peptide abundance in the final collagen hydrolysate, particularly an increased presence of tripeptides (composed of three amino acids), could be translated into enhanced multifunctional bioactivities, specifically assessing and comparing the capacity of the collagen hydrolysates to inhibit ACE and DPP-IV activities and to stimulate GLP-1 secretion at the cellular level.

The results presented herein demonstrate a superior biological activity of the tripeptide-enriched formulation, supporting its potential application as a functional ingredient for integrated health support. 

## 2. Materials and Methods

### 2.1. Chemicals

Reagents and chemicals used in this study were of analytical grade and commercially available. Full details are reported in the Supplementary Materials. CH was provided by Sel Sanayi Ürünleri Ticaret ve Pazarlama A.Ş. Beyoğlu, İstanbul, Türkiye.

### 2.2. In Vitro Angiotensin Converting Enzyme (ACE) Inhibition Assay

In vitro ACE inhibitory activity was assessed using the HHL assay as previously reported; full experimental details are provided in the Supplementary Materials [9].

### 2.3. In Vitro Dipeptidil-Peptidase-IV (DPP-IV) Inhibition Assay

DPP-IV inhibitory activity was assessed using a fluorescence-based assay (Cayman Chemical Company, Ann Arbor, MI, USA), as detailed in the Supplementary Materials. Samples had final concentrations of 0.5, 1.0, 5.0 or 10.0 mg/mL.

### 2.4. Cell Culture Conditions

Human intestinal Caco-2 cells (INSERM, Paris, France) and murine enteroendocrine STC-1 cells (ATCC HB-8065; LGC Standards, Milan, Italy) were cultured under standard conditions at 37 °C in a humidified atmosphere containing 5% CO_2_. Cells were grown in high-glucose DMEM (25 mM) supplemented with 3.7 g/L sodium bicarbonate, 4 mM stabilized L-glutamine, 1% non-essential amino acids, 100 U/L penicillin, and 100 μg/L streptomycin, and enriched with 10% heat-inactivated fetal bovine serum. Routine subculturing was performed upon reaching appropriate confluence.

### 2.5. Cellular DPP-IV Inhibition Assay

Caco-2 cellular DPP-IV inhibition was assessed following sample incubation (10 mg/mL), using a fluorogenic substrate assay, with full experimental details provided in the Supplementary Materials.

### 2.6. Enzyme-Linked Immunosorbent Assays (ELISAs) for GLP-1 Quantification in Cell Culture Supernatants

STC-1 cellular GLP-1 secretion was evaluated following incubation with samples (10 mg/mL) using a commercial ELISA kit (Millipore, Watford, UK). Cells were treated with either vehicle or sample, and secreted GLP-1 was quantified according to the manufacturer’s protocol, with full experimental details provided in the Supplementary Materials.

### 2.7. Degree of Hydrolysis (DH) of the Hydrolysates

The degree of hydrolysis (DH) of CHand BCH samples was assessed using the o-phthaldialdehyde (OPA) assay, based on the reaction of α-amino groups with the OPA reagent. Samples were incubated with the reagent and absorbance was measured, as previously described [10]. Full experimental details are provided in the Supplementary Materials.

### 2.8. High-Resolution Mass Spectrometry Analysis (nLC-HRMS)

All samples were desalted, reconstituted, and analyzed by nano-liquid chromatography coupled to high-resolution Orbitrap mass spectrometry to profile peptide mixtures. Detailed procedures, including LC gradients, columns, and MS settings, are provided in the Supplementary Materials.

### 2.9. Statistical Analysis

All data sets were analyzed using One-way Anova followed by Tukey’s post hoc analysis, or Two-way ANOVA followed by Sidak’s multiple comparison test (GraphPad Software 9, San Diego, CA, USA). Values were expressed as means ± standard deviation; *p*-values ≤ 0.05 were considered significant. Proteomics data were statistically analyzed using PLS-DA in MetaboAnalyst v 6.0 (https://www.metaboanalyst.ca/) accessed on date 11 February 2026.

## 3. Results

### 3.1. In Vitro Inhibitory Activity of Collagen Hydrolysates on Angiotensin-Converting Enzyme (ACE)

**Figure 1:**
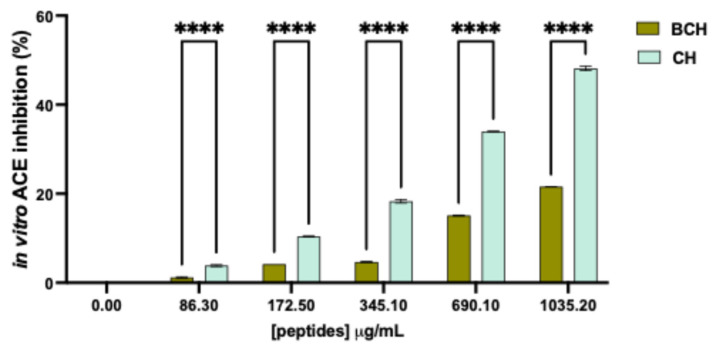
Results of ACE enzyme inhibition by CH and BCH, tested at different concentrations (µg/mL); Data represent the mean ± s.d. of three determinations performed in triplicate. All data sets were analyzed using Two-way ANOVA. (****) *p* < 0.0001. BCH: Collagen hydrolysate benchmark; CH: CollaSel TRIPEPTIDE.

The inhibitory activity of the peptides on the ACE enzyme, which is involved in blood pressure regulation mechanisms, was evaluated in vitro. The results in Figure 1 show that the BCH and CH samples exhibit dose-dependent ACE inhibitory activity in the tested concentration range of 86.3 to 1035.2 µg/mL. Specifically, the CH peptides were significantly more active than the BCH peptides when tested at the same concentration in inhibiting ACE in vitro, with a maximum inhibition percentage of 21.59 ± 0.01% for BCH and 48.18 ± 0.50% for CH.

### 3.2. CH Efficiently Inhibits In Vitro Dipeptidyl Peptidase-IV (DPP-IV) Enzyme

**Figure 2:**
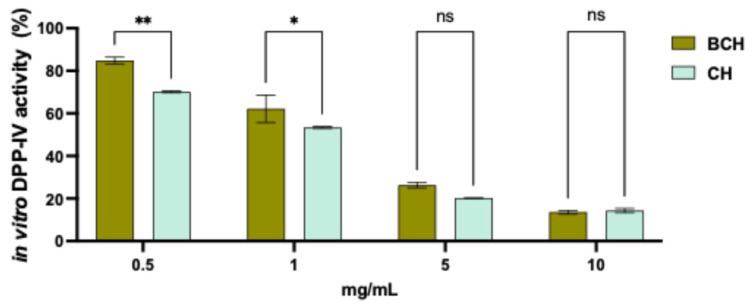
Results of the residual activity of the DPP-IV enzyme following treatment with CH and BCH, tested at different concentrations (mg/mL). Data represent the mean ± s.d. of three determinations performed in triplicate. All data sets were analyzed using Two-way ANOVA. (*) *p* < 0.05, (**) *p* < 0.01, ns: not significant. BCH: Collagen hydrolysate benchmark; CH: CollaSel TRIPEPTIDE.

To evaluate the inhibitory activity of CH and BCH peptides on the DPP-IV enzyme, a fluorescence-based in vitro assay was used. DPP-IV is responsible for the degradation of GLP-1, an incretin hormone that stimulates insulin secretion in a glucose-dependent manner and inhibits glucagon secretion; its prolonged action contributes to better blood glucose control. The aim of the study was therefore to investigate the potential inhibitory effect of collagen-derived peptides on DPP-IV activity. The results (Figure 2) show that CH and BCH peptides inhibit DPP-IV enzymatic activity in a dose-dependent manner within a tested concentration range of 0.5 to 10.0 mg/mL. Specifically, BCH peptides inhibited DPP-IV activity by 15.14 ± 1.59%, 37.83 ± 6.47%, 73.67 ± 1.26%, 86.41 ± 0.88%, and 89.82 ± 0.98% at concentrations of 0.5, 1.0, 5.0, 10.0, and 20.0 mg/mL, respectively. In comparison, CH peptides inhibited DPP-IV by 29.87 ± 0.45%, 46.61 ± 0.45%, 79.74 ± 0.01%, 85.61 ± 0.98%, and 84.06 ± 1.61% at the same concentrations, respectively. The data show that at the tested concentrations of 0.5 and 1.0 mg/mL, CH peptides are statistically more active than BCH peptides, suggesting a higher hypoglycemic effect of BCH. These findings are consistent with the data obtained from the ACE inhibition assay.

### 3.3. Evaluation of CH Effect on Caco-2 and STC-1 Cell Line Viability

**Figure 3:**
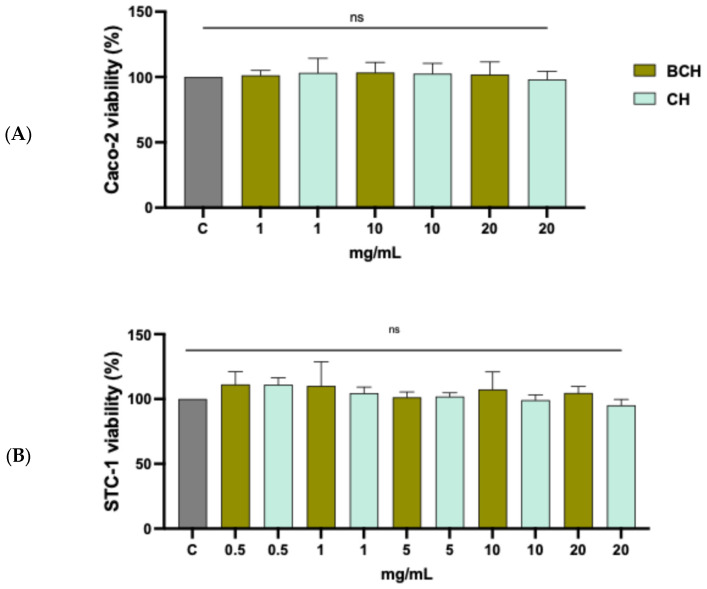
Results on the viability of cells treated with BCH and CH. MTT results on (**A**) Caco-2 and (**B**) STC-1 cells. Cells were treated within the range of 1–20 mg/mL. Data represent the mean ± s.d. of three determinations performed in triplicate All data sets were analyzed using one-way ANOVA. ns: not statistically significant. C: control cells; BCH: Collagen hydrolysate benchmark; CH: CollaSel TRIPEPTIDE.

To investigate the effects of the collagen peptides BCH and CH in the intestinal cellular system, Caco-2 (human intestinal) and STC-1 (murine enteroendocrine) cells were used. Specifically, to evaluate the cellular compatibility of the two samples with both cell systems, an MTT assay (3-(4,5-dimethylthiazol-2-yl)-2,5-diphenyltetrazolium bromide) was performed. This is a standard colorimetric test for measuring mitochondrial enzymatic activity, which reduces MTT to formazan, giving the substance a blue/purple coloration. Caco-2 and STC-1 cells were treated with increasing concentrations of the BCH and CH compounds for 24 h and 2 h, respectively, to assess their effect on cell viability. Following treatment, the MTT solution (5 mg/mL in phosphate-buffered saline) was added to the cells and incubated for 2 h. After incubation, the solution was removed, the cells were lysed, and the absorbance of the formazan was measured at a wavelength of 570 nm. The results (Figure 3) showed that both BCH and CH compounds were non-cytotoxic to Caco-2 and STC-1 cells up to a concentration of 20 mg/mL, indicating good cellular tolerability and allowing the continuation of subsequent experiments under conditions compatible with cell viability.

### 3.4. Evaluation of In Situ CH Inhibition of DPP-IV Enzyme in Caco-2 Intestinal Cells

**Figure 4:**
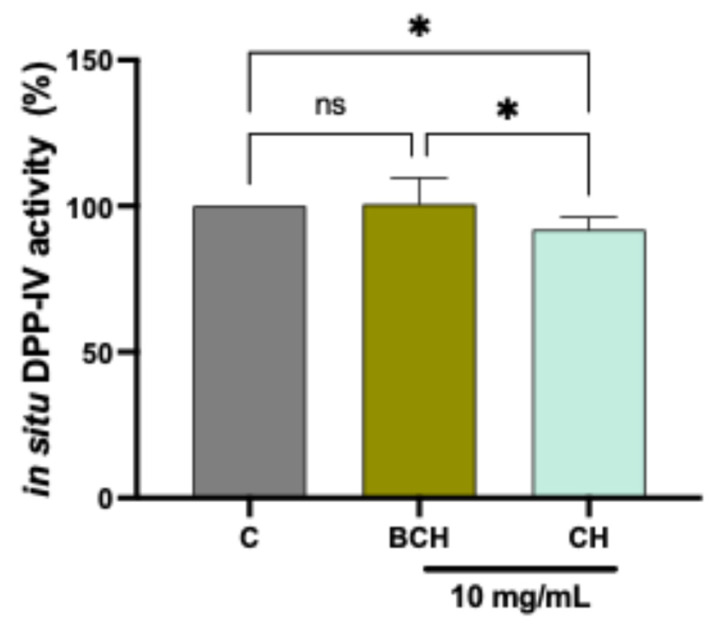
Results of residual cellular DPP-IV enzyme activity following treatment with CH and BCH peptides at 10 mg/mL. Data represent the mean ± s.d. of three determinations performed in triplicate. All data sets were analyzed using one-way ANOVA. (*) *p* < 0.05, ns: not statistically significant. C: control cells; BCH: Collagen hydrolysate benchmark; CH: CollaSel TRIPEPTIDE.

To evaluate the effectiveness of DPP-IV enzyme inhibition by BCH and CH in a physiologically relevant cellular context, an in situ assay was conducted using Caco-2 intestinal cells, which express this enzyme in its transmembrane form. This approach allows for the study of enzymatic activity within a cellular microenvironment that closely mimics intestinal conditions, providing a more accurate assessment of the compounds’ potential efficacy. The results (Figure 4) show that the CH peptides, tested at a concentration of 10 mg/mL at the cellular level, significantly inhibited enzymatic activity compared to untreated control cells (a reduction of 8.12 ± 4.25%), unlike the BCH peptides, which did not significantly reduce enzymatic activity at the cellular level. Therefore, CH proved to be more effective in inhibiting the enzyme expressed by the cells compared to BCH. Although the peptides showed marked efficacy in inhibiting DPP-IV enzymatic activity in the purified in vitro system, this activity was reduced when tested in situ on Caco-2 cells. This discrepancy could be attributed to various factors, including possible degradation by extracellular peptidases or inefficient access to the active site of the transmembrane DPP-IV.

### 3.5. Assessment of the CH Ability to Modulate GLP-1 Hormone Secretion in STC-1 Enteroendocrine Cells

**Figure 5:**
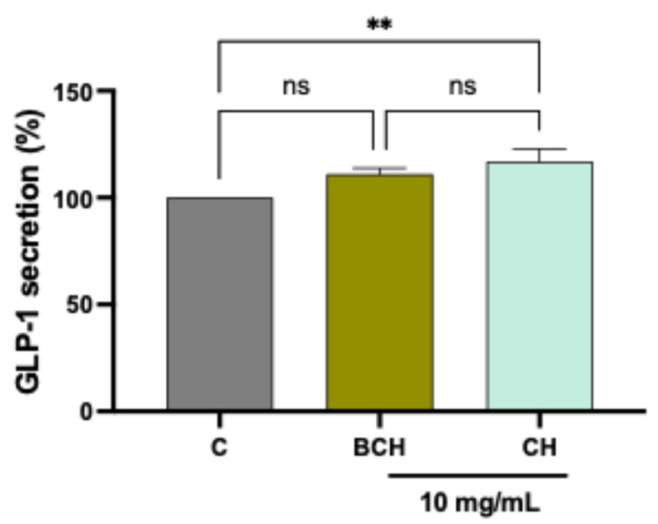
Results of GLP-1 hormone secretion by cells treated with CH and BCH at 10 mg/mL. Data represent the mean ± s.d. of three determinations performed in triplicate. All data sets were analyzed using one-way ANOVA. (**) *p* < 0.01, ns: not statistically significant. C: control cells; BCH: Collagen hydrolysate benchmark; CH: CollaSel TRIPEPTIDE.

The STC-1 cell line, derived from a secretin-secreting intestinal tumor, shares many characteristics with native intestinal enteroendocrine cells. For this reason, it is widely used as an in vitro model for screening foods or compounds capable of modulating gastrointestinal hormone secretion. The release of satiety hormones by enteroendocrine cells, such as GLP-1 produced by intestinal L cells, plays a crucial role in regulating energy balance and glycemic control. In this study, we analyzed the effect of BCH and CH compounds on GLP-1 secretion by STC-1 cells using a biochemical ELISA assay. The results in Figure 5 indicate that CH peptides significantly stimulate GLP-1 release after 1 h of treatment (an increase of 16.9 ± 5.88% compared to untreated control cells), unlike BCH peptides, where GLP-1 production remains comparable to the control condition (+11 ± 2.8%, statistically not significant).

### 3.6. Proteomic Profile of Collagen Hydrolysates

**Figure 6:**
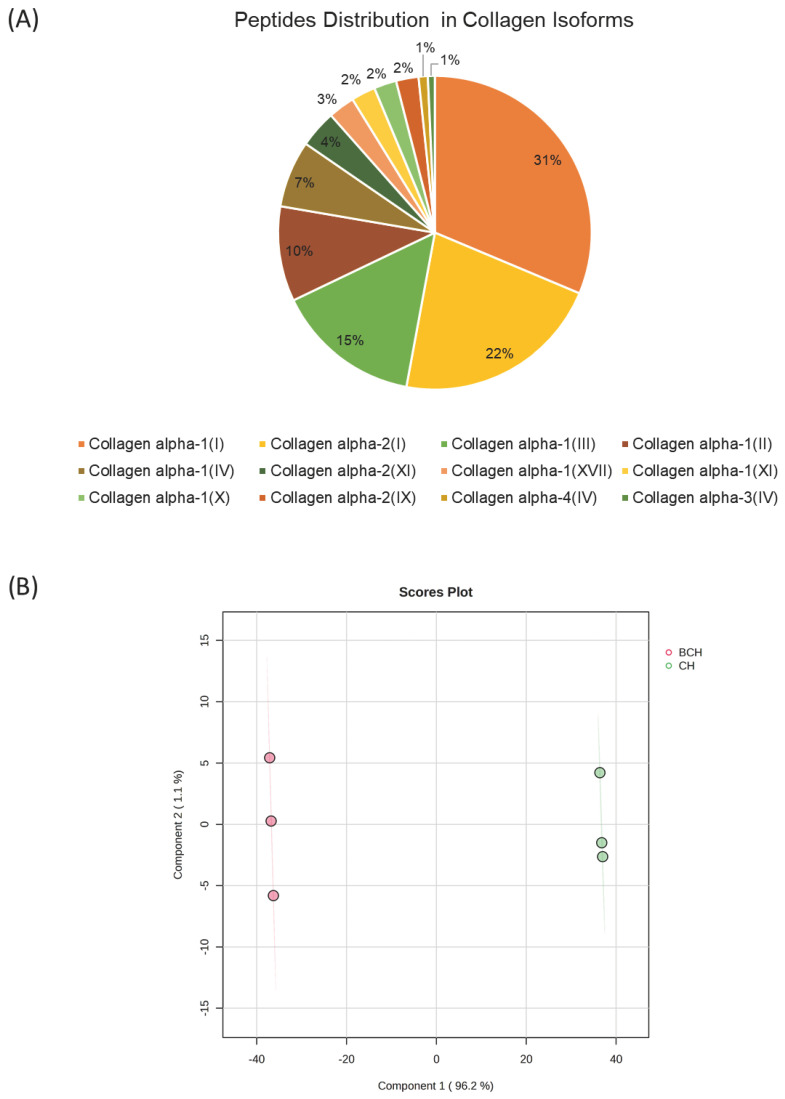
(**A**) Abundance of Collagen isoforms identified in CH and BCH. BCH: Collagen hydrolysate benchmark; CH: CollaSel TRIPEPTIDE. (**B**) Partial Least Squares Discriminant Analysis (PLS-DA) score plot of the proteomic profiles obtained by nanoLC–HRMS for CH and BCH samples (*n* = 3 instrumental replicates per group).

Proteomic profiling revealed a collagen-rich peptide landscape, with 1683 peptides confidently assigned to collagen isoforms. In this workflow, only peptides of ≥4 amino acids were retained for protein inference, ensuring reliable mapping to the corresponding collagen chains. As summarized in Table 1 and visualized in Figure 6, peptide identifications were unevenly distributed across isoforms and strongly dominated by fibrillar collagens: collagen alpha-1(I) yielded 527 peptides (31.3%), collagen alpha-2(I) 363 peptides (21.6%), and collagen alpha-1(III) 252 peptides (15.0%), together accounting for ~68% of all collagen-assigned peptides. Additional contributions were observed for collagen alpha-1(II) (165 peptides) and collagen alpha-1(IV) (116 peptides), whereas minor isoforms such as collagen alpha-4(IV) (16 peptides) and collagen alpha-3(IV) (12 peptides) represented <1% each, indicating substantially lower peptide recovery for these chains under the applied conditions. Beyond the major fibrillar components, peptides were also detected from structurally specialized collagens, including XI (alpha-2 and alpha-1 chains), IX (alpha-2), X (alpha-1), and XVII (alpha-1), supporting the presence of a broader collagen repertoire in the sample rather than a profile restricted to type I collagen alone. From a statistical point, a clear separation between CH (green) and BCH (pink) was observed along Component 1, which explained 96.2% of the total variance, indicating distinct peptide composition patterns between the two hydrolysates. Component 2 accounted for 1.1% of the variance. The tight clustering of replicates within each group confirms good analytical reproducibility and supports the robustness of the multivariate model (Figure 6B).

**Figure 7:**
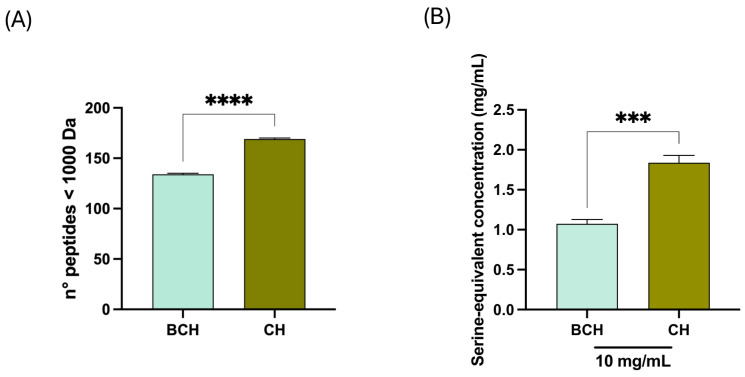
(**A**) N° peptides < 1000 Da for BCH andCH; (**B**) Degree of hydrolysis of BCH and CH, respectively. All data sets were analyzed using *t*-student. (***) *p* < 0.001; (****) *p* < 0.0001; BCH: Collagen hydrolysate benchmark; CH: CollaSel TRIPEPTIDE.

Peptide size profiling further clarified differences between the two samples BCH and CH. The molecular weight distribution (Figure 7A) showed that, in both cases, the peptide population was concentrated in the intermediate MW range (1000–2000 Da), consistent with a predominance of moderately sized collagen fragments. However, the datasets diverged in their relative enrichment of short versus long species: CH contained 169 peptides ≤ 1000 Da (16.78%), 657 peptides between 1000–2000 Da (65.24%), and 181 peptides > 2000 Da (17.97%), whereas BCH contained 134 peptides ≤ 1000 Da (10.41%), 719 peptides between 1000–2000 Da (55.87%), and 434 peptides > 2000 Da (33.72%). Thus, compared with CH, the BCH dataset displayed a marked shift toward higher-MW peptides, nearly doubling the proportion of >2000 Da fragments. This pattern is also consistent with the binned distribution shown in Figure 7A, where BCH shows less amount of low molecular weight (LMW) peptide compared to CH. In particular, focusing the analysis on small-chain peptides, data reported in detail in Table 2, clearly suggest that CH contains 14.2% of LMW peptides, while BCH contains 8.5% of LMW peptides, demonstrating that CH contains 67% more small peptides than BCH, being thus enriched in the peptide fraction between 850 and 1000 Da.

Physicochemical descriptors were highly consistent between CH and BCH. The aliphatic index was 30.23 (BCH) and 30.37 (CH), while the GRAVY scores were strongly negative (−0.769 and −0.772, respectively), indicating that both peptide pools were predominantly hydrophilic and exhibited nearly overlapping global hydropathy signatures. Overall, the combined isoform distribution, MW stratification, and residue composition converge on a coherent result: the peptidome is dominated by canonical collagen fragments, with BCH capturing a substantially larger fraction of longer peptides, while CH is relatively enriched in medium-sized collagen peptides, without major differences in bulk hydrophobicity or collagen-typical amino acid content. In line with these results and Figure 7A, the OPA assay results (Figure 7B) indicate that CH exhibits a higher degree of hydrolysis than BCH (1.839 ± 0.09% and 1.073 ± 0.05%, respectively). These data suggest that CH is characterized by a higher content of free amino acids. When expressed as a ratio, CH exhibited a 1.7-fold higher free amino group content than BCH, confirming a higher degree of hydrolysis. The higher degree of hydrolysis of CH compared to the benchmark (BCH) reinforces the molecular weight distribution data, favoring an enrichment of peptides in CH sample compared to BCH. In fact, a higher OPA value is the consequence of an increased breaking of peptide bonds within the starting proteins, leading to greater peptide release in the final product.

## 4. Discussion

A growing body of evidence indicates that protein hydrolysates can simultaneously modulate key enzymatic systems, cellular signaling pathways, and hormone secretion mechanisms. For example, several protein hydrolysates have been reported to exert antihypertensive effects through inhibition of angiotensin-converting enzyme (ACE), while also influencing glucose homeostasis via modulation of dipeptidyl peptidase IV (DPP-IV) activity and incretin signaling. Importantly, these effects are often mediated by short peptides, including di- and tripeptides [11]. The multifunctionality of protein hydrolysates is particularly relevant in the context of complex, multifactorial disorders such as hypertension and type 2 diabetes mellitus, where dysregulation of interconnected pathways—including the renin–angiotensin system, endothelial function, incretin hormones, and low-grade inflammation—contributes to disease progression. Nutritional interventions capable of targeting more than one of these mechanisms may therefore offer advantages over single-target approaches, especially in early or subclinical stages of disease. 

Among food-derived protein hydrolysates, collagen hydrolysates have emerged as particularly promising candidates for multifunctional health applications. In addition to their well-established roles in skin, joint, and connective tissue health, collagen-derived peptides have been increasingly associated with cardiometabolic benefits, including blood pressure modulation and improvements in glucose regulation. Moreover, several randomized and controlled human studies have demonstrated that specific collagen hydrolysates can improve parameters related to glucose homeostasis, vascular function, and blood pressure regulation.

The present study provides a comprehensive biochemical, cellular, and proteomic characterization of a collagen hydrolysate named CH, highlighting its differential potential in supporting human health. Collectively, the results demonstrate that the tripeptide-enriched formulation (CollaSel TRIPEPTIDE, CH) exerts superior biological activity compared with the collagen hydrolysate benchmark (BCH), particularly in pathways relevant to blood pressure regulation and glucose homeostasis, while maintaining an excellent safety profile at the cellular level. From a cardiovascular standpoint, the pronounced ACE-inhibitory activity observed for both hydrolysates supports the growing body of evidence indicating that food-derived peptides can act as natural modulators of the renin–angiotensin system. ACE inhibition is a well-established therapeutic strategy for hypertension management, and several studies have demonstrated that collagen-derived peptides rich in proline and hydroxyproline residues effectively interact with the ACE catalytic site, leading to antihypertensive effects in vivo [4]. 

In the present study, CH exhibited nearly double the ACE inhibitory activity of BCH at equivalent concentrations, suggesting that enrichment in short peptides enhances functional potency. This observation is consistent with previous reports indicating that low-molecular-weight peptides display improved ACE inhibition and higher intestinal bioavailability compared with longer peptide fragments [3]. Clinical evidence also supports the antihypertensive potential of collagen hydrolysates. In subjects with mild or high-normal blood pressure, daily intake of chicken collagen hydrolysate (2.9 g/day) for 18 weeks resulted in significant reductions in systolic blood pressure and arterial stiffness, as assessed by brachial–ankle pulse wave velocity, alongside increased nitric oxide bioavailability [12]. Similarly, short-term supplementation with chicken collagen hydrolysate in individuals with pre- or mild hypertension produced clinically meaningful reductions in blood pressure and plasma renin activity, suggesting modulation of the renin–angiotensin system in humans [13].

In parallel, both collagen hydrolysates demonstrated a robust, dose-dependent inhibitory effect on DPP-IV activity, an enzyme responsible for the rapid inactivation of incretin hormones such as GLP-1. DPP-IV inhibition is a validated pharmacological approach for improving glycemic control in type 2 diabetes, primarily through prolongation of endogenous GLP-1 action [6]. Notably, CH showed significantly greater inhibitory activity than BCH at lower concentrations, suggesting a higher intrinsic hypoglycemic potential. These findings align with previous studies demonstrating that food protein–derived peptides can effectively inhibit DPP-IV and contribute to improved glucose metabolism [11]. Importantly, the functional relevance of DPP-IV inhibition was further supported by experiments conducted in Caco-2 intestinal cells, which express DPP-IV in its membrane-bound form. While both hydrolysates were active in the purified enzymatic assay, only CH significantly reduced cellular DPP-IV activity. Nonetheless, the retention of significant activity highlights the translational relevance of the CH-enriched formulation. 

Notably, given the dynamic nature of the cellular model, where small peptides are rapidly absorbed and metabolized, it was essential to test a concentration sufficiently high to ensure measurable bioactivity at the cellular level. Importantly, unlike cell-free enzymatic assays, the in situ human intestinal Caco-2 model represents a dynamic biological system in which peptides may interact dynamically with the cellular brush border peptidases, being continuously absorbed, and potentially degraded. Therefore, the effective concentration at the site of the membrane-bound DPP-IV enzyme is likely lower than the nominal concentration applied, leading to a loss in collagen hydrolysates bioactivity compared to the in vitro experiments. Interestingly, these results are in line with previous literature evidence supporting this behavior [14,15].

Beyond enzyme inhibition, CH uniquely stimulated GLP-1 secretion in STC-1 enteroendocrine cells, indicating a dual mechanism of action combining incretin preservation and enhanced hormone release. The ability of food-derived peptides to stimulate GLP-1 secretion has been previously reported for milk- and fish-derived peptides and is considered highly relevant for metabolic health [16]. GLP-1 plays a central role not only in glucose-dependent insulin secretion but also in appetite regulation, endothelial function, and cardiovascular protection [17]. Therefore, the observed increase in GLP-1 release further supports the potential of CH as a multifunctional ingredient targeting multiple aspects of metabolic homeostasis. In line with these in vitro results, a recent proof-of-concept clinical study demonstrated that oral supplementation with a specifically designed collagen hydrolysate (H80, 5 g per dose) significantly reduced postprandial glucose excursions in healthy, normoglycemic, and prediabetic individuals, an effect associated with increased circulating GLP-1 levels [18]. These findings closely mirror the dual mechanism observed in the present study for the tripeptide-enriched formulation, combining DPP-IV inhibition with stimulation of GLP-1 secretion, and support the translational relevance of collagen-derived peptides in glycemic control. Although both hydrolysates were dominated by canonical collagen fragments, dedicated analysis reveals that CH was relatively enriched in short- and medium-length peptides, whereas BCH contained a higher proportion of long peptides (>2000 Da). Short peptides are generally associated with improved intestinal absorption and enhanced interaction with molecular targets such as ACE and DPP-IV [19]. This size-dependent effect likely contributes to the superior bioactivity of CH observed across multiple assays. Importantly, human pharmacokinetic studies have demonstrated that enzymatically hydrolyzed collagen is rapidly absorbed, leading to increased plasma levels of collagen-specific amino acids and bioactive di- and tripeptides, including hydroxyproline-containing motifs [20]. This supports the notion that peptide size and hydrolysis strategy, key discriminating factors between the two formulations examined in the present study, are critical determinants of bioavailability and, ultimately, clinical efficacy. Crucially, both collagen hydrolysates displayed excellent cellular tolerability in intestinal and enteroendocrine cell models, with no cytotoxic effects observed even at high concentrations. This safety profile is essential for the development of collagen-derived peptides as nutraceuticals or functional food ingredients intended for long-term consumption.

This study provides promising in vitro evidence supporting the beneficial bioactivities of highly hydrolyzed collagen hydrolysates, particularly those enriched in low–molecular weight peptides. However, further in vivo investigations and well-designed clinical studies are required to establish the physiological relevance of these findings. Such studies will be essential to confirm the translational potential of the observed effects and to contextualize the results within the existing body of literature on the bioactivity of collagen hydrolysates.

## 5. Conclusions

By applying a multidisciplinary and advanced in vitro and cellular techniques, the study provides molecular evidence regarding the ability of collagen hydrolysate to selectively target key enzymes involved in the hypertension and hyperglycemia progression. 

Our findings are in line with previous reports indicating that collagen supplementation may represent a promising nutritional strategy for the management of metabolic syndrome (MetS)-related risk factors [21], thus highlighting their physiological relevance. In this context, both BCH and CH demonstrated dose-dependent inhibition of ACE and DPP-IV activity in vitro, confirming their potential to modulate key enzymatic targets involved in blood pressure regulation and glucose homeostasis.

Importantly, CH exhibited greater potency and retained biological activity in a physiologically relevant cellular model, where it significantly inhibited membrane-bound DPP-IV and stimulated GLP-1 secretion. The use of intestinal cell models strengthens the translational relevance of our data, as these systems better reflect the dynamic environment in which dietary peptides interact with enterocytes, undergo absorption, and modulate metabolic pathways in situ. 

Collectively, these findings support the concept that collagen hydrolysates may contribute to the nutritional modulation of pathways implicated in MetS. Importantly, in this scenario, our results suggest that a higher degree of hydrolysis, leading to enrichment in low-molecular-weight peptides, may enhance biological efficacy, likely due to improved bioavailability and interaction with enzymatic targets.

## Figures and Tables

**Table 1:** Collagen isoforms identified in CH and BCH.

Accession	Description	Peptides (*n*°)
P02453	Collagen alpha-1(I)	527
P02465	Collagen alpha-2(I)	363
P04258	Collagen alpha-1(III)	252
P02459	Collagen alpha-1(II)	165
Q7SIB2	Collagen alpha-1(IV)	116
Q32S24	Collagen alpha-2(XI)	65
A6QPB3	Collagen alpha-1(XVII)	46
Q28083	Collagen alpha-1(XI)	42
P23206	Collagen alpha-1(X)	39
C0HLN2	Collagen alpha-2(IX)	39
Q29442	Collagen alpha-4(IV)	16
Q28084	Collagen alpha-3(IV)	12

**Table 2:** Number of Low Molecular Weight (LMW) Peptide Distribution in CH and BCH. BCH: Collagen hydrolysate benchmark; CH: CollaSel TRIPEPTIDE.

	Small (≤850 Da)	Medium (850–950 Da)	Long (950–1000 Da)	Tot. LWM Peptides (<1000)	
CH	43	77	48	169	14.2% (+67% vs. BCH)
BCH	38	59	36	133	8.5%

## Data Availability

The raw data supporting the conclusions of this article will be made available by the authors on request.

## Supplementary Materials

The following supporting information can be downloaded at: https://www.mdpi.com/article/10.3390/biomedicines14030589/s1.

## Abbreviations

ACE	Angiotensin-Converting Enzyme
DPP-IV	Dipeptidyl Peptidase IV
GLP-1	Glucagon-Like Peptide-1
T2DM	Type 2 Diabetes Mellitus
RAS	Renin–Angiotensin System
CH	CollaSel TRIPEPTIDE (Tripeptide-Enriched Collagen Hydrolysate)
BCH	Benchmark Collagen Hydrolysate
BCH	Collagen Hydrolysate Benchmark
DMEM	Dulbecco’s Modified Eagle’s Medium
FBS	Fetal Bovine Serum
ELISA	Enzyme-Linked Immunosorbent Assay
OPA	o-Phthaldialdehyde
nanoLC–HRMS	Nano Liquid Chromatography–High-Resolution Mass Spectrometry
HHL	Hippuryl-Histidyl-Leucine
HA	Hippuric Acid
HPLC	High-Performance Liquid Chromatography
MTT	3-(4,5-Dimethylthiazol-2-yl)-2,5-Diphenyltetrazolium Bromide
DH	Degree of Hydrolysis
MW	Molecular Weight
GRAVY	Grand Average of Hydropathy

## References

[1] Proksch E., Segger D., Degwert J., Schunck M., Zague V., Oesser S. (2014). Oral Supplementation of Specific Collagen Peptides Has Beneficial Effects on Human Skin Physiology: A Double-Blind, Placebo-Controlled Study. Ski. Pharmacol. Physiol..

[2] Ahmad H., Khan H., Haque S., Ahmad S., Srivastava N., Khan A. (2023). Angiotensin-Converting Enzyme and Hypertension: A Systemic Analysis of Various ACE Inhibitors, Their Side Effects, and Bioactive Peptides as a Putative Therapy for Hypertension. J. Renin Angiotensin Aldosterone Syst..

[3] Iwaniak A., Minkiewicz P., Darewicz M. (2014). Food-Originating ACE Inhibitors, Including Antihypertensive Peptides, as Preventive Food Components in Blood Pressure Reduction. Compr. Rev. Food. Sci. Food Saf..

[4] Mizutani K., Ikeda K., Ishikado A., Kawai Y., Yamori Y. (2000). Antihypertensive Effect of Cattle Bone Collagen-Derived Peptides in Ovariectomized Stroke-Prone Spontaneously Hypertensive Rats. Clin. Exp. Pharmacol. Physiol..

[5] Green B.D., Flatt P.R., Bailey C.J. (2006). Dipeptidyl Peptidase IV (DPP IV) Inhibitors: A Newly Emerging Drug Class for the Treatment of Type 2 Diabetes. Diabetes Vasc. Dis. Res..

[6] Holst J.J., Deacon C.F. (2005). Glucagon-like Peptide-1 Mediates the Therapeutic Actions of DPP-IV Inhibitors. Diabetologia.

[7] Wu P.-Y., Hsieh C.-H., Iqbal A., Lin Y.-S., Cheng M.-W., Chang L.-H., Huang S.-M., Hsu K.-C. (2025). Bioactive Peptides from Sodium Caseinate Hydrolysate with High Oral Absorption Regulate Blood Glucose in Type 2 Diabetic Mice via Inhibition of DPP-IV and Stimulation of GLP-1. Foods.

[8] Han R., Tian J., Han Y., Wang G., Zhou G., Dai C., Wang C. (2025). Crucian Carp-Derived ACE-Inhibitory Peptides with In Vivo Antihypertensive Activity: Insights into Bioactivity, Mechanism, and Safety. Molecules.

[9] Boschin G., Scigliuolo G.M., Resta D., Arnoldi A. (2014). ACE-Inhibitory Activity of Enzymatic Protein Hydrolysates from Lupin and Other Legumes. Food Chem..

[10] Nielsen P.M., Petersen D., Dambmann C. (2001). Improved Method for Determining Food Protein Degree of Hydrolysis. J. Food Sci..

[11] Power O., Nongonierma A.B., Jakeman P., FitzGerald R.J. (2014). Food Protein Hydrolysates as a Source of Dipeptidyl Peptidase IV Inhibitory Peptides for the Management of Type 2 Diabetes. Proc. Nutr. Soc..

[12] Kouguchi T., Ohmori T., Shimizu M., Takahata Y., Maeyama Y., Suzuki T., Morimatsu F., Tanabe S. (2013). Effects of a Chicken Collagen Hydrolysate on the Circulation System in Subjects with Mild Hypertension or High-Normal Blood Pressure. Biosci. Biotechnol. Biochem..

[13] Saiga-Egusa A., Iwai K., Hayakawa T., Takahata Y., Morimatsu F. (2009). Antihypertensive Effects and Endothelial Progenitor Cell Activation by Intake of Chicken Collagen Hydrolysate in Pre- and Mild-Hypertension. Biosci. Biotechnol. Biochem..

[14] Taglioni E., Aita S.E., Bollati C., Boschin G., Cavaliere C., d’Adduzio L., Montone C.M., Laganá A., Lammi C., Capriotti A.L. (2025). Seaweed (*G. gracilis*) Protein Hydrolyzates: A Valuable Source of Short- and Medium-Chain Peptides with Multifunctional Properties. J. Agric. Food Chem..

[15] Kotsoni E., Daukšas E., Hansen Aas G., Rustad T., Tiwari B.K., Lammi C., Bollati C., Fanzaga M., d’Adduzio L., Stangeland J.K. (2024). Antioxidant Activity and DPP-IV Inhibitory Effect of Fish Protein Hydrolysates Obtained from High-Pressure Pretreated Mixture of Rainbow Trout (*Oncorhynchus mykiss*) and Atlantic Salmon (*Salmo salar*) Rest Raw Material. Mar. Drugs.

[16] Vivanco-Maroto S.M., Gómez-Marín C., Recio I., Miralles B. (2025). Milk Peptides Found in Human Jejunum Induce Enteroendocrine Hormone Secretion and Inhibit DPP-IV. Food Funct..

[17] Nadkarni P., Chepurny O.G., Holz G.G. (2014). Regulation of Glucose Homeostasis by GLP-1. Progress in Molecular Biology and Translational Science.

[18] Grasset E., Briand F., Virgilio N., Schön C., Wilhelm M., Cudennec B., Ravallec R., Aboubacar H., Vleminckx S., Prawitt J. (2024). A Specific Collagen Hydrolysate Improves Postprandial Glucose Tolerance in Normoglycemic and Prediabetic Mice and in a First Proof of Concept Study in Healthy, Normoglycemic and Prediabetic Humans. Food Sci. Nutr..

[19] Sasaoka Y., Takagi T., Michiba S., Yamamoto Y., Kumagai Y., Kishimura H. (2021). Study on the Mechanism of the Blood-Glucose-Lowering Effect of Collagen Peptides from Sturgeon By-Products. Mar. Drugs.

[20] Skov K., Oxfeldt M., Thøgersen R., Hansen M., Bertram H.C. (2019). Enzymatic Hydrolysis of a Collagen Hydrolysate Enhances Postprandial Absorption Rate—A Randomized Controlled Trial. Nutrients.

[21] Pueyo-Arias M., López-Yoldi M., Navas-Carretero S., González-Navarro C.J., Zulet M.D.L.Á., Milagro F.I. (2026). Collagen Supplementation in Metabolic Syndrome: A Narrative Review Unraveling the Biological Mechanisms and Effects. Nutr. Res. Rev..

