# Supplementary material for: In Vitro Evaluation of ACE and DPP-IV Inhibitory, and GLP-1 Stimulation Activities of Collagen Hydrolysate Enriched in Tripeptides
Source: Biomedicines. 2026 Mar 5;14(3):589. doi: 10.3390/biomedicines14030589 (PMC13023980; doi:10.3390/biomedicines14030589)
Supplement: Supplementary file 1 [file biomedicines-14-00589-s001.zip › biomedicines-4179346-supplementary.pdf]

biomedicines

MDPI

Supplementary Materials

In Vitro Evaluation of ACE and DPP-IV Inhibitory, and GLP-1 Stimulation Activities of Collagen Hydrolysate Enriched in Tripeptides

Melissa Fanzaga [1], Lorenza d'Adduzio [1], Carlotta Bollati [1], Maria Silvia Musco [1], Giovanna Boschin [1], Gilda Aiello [2] and Carmen Lammi [1,*]

[1] Department of Pharmaceutical Sciences, University of Milan, 20133 Milan, Italy
[2] Department of Human Science and Quality of Life Promotion, Telematic University San Raffaele, 00166 Rome, Italy
* Correspondence: carmen.lammi@unimi.it; Tel.:+39-02-50319372:

S1. Materials and Methods

S1.1. Chemicals

All chemicals and reagents employed in this study were of analytical grade and commercially obtainable. Dulbecco's Modified Eagle's Medium (DMEM), L-glutamine, fetal bovine serum (FBS), phosphate-buffered saline (PBS), penicillin/streptomycin and multiwell plates were sourced from Euroclone (Milan, Italy). The (GLP-1) ELISA Kit (Catalog No. EZGLP1T-36K) and porcine ACE were supplied by Sigma-Aldrich (St. Louis, MO, USA).

S1.2. In Vitro Angiotensin Converting Enzyme (ACE) Inhibition Assay

In vitro ACE inhibitory activity was evaluated by measuring the formation of hippuric acid (HA) from hippuryl-histidyl-leucine (HHL), a mimic substrate for ACE I, as previously reported [1]. Briefly, 100 μL of HHL (2.5 mmol L^{-1}) in tris-formic acid (100 mmol L^{-1}), NaCl (300 mmol L^{-1}) at pH 8.3 (buffer 1) was mixed with 30 μL of sample in the same buffer at five concentrations (186, 173, 345, 690, 1035 μg mL^{-1}). Samples were preincubated at 37 °C for 15 min, then 15 μL of 1.67×10^{-3} nkat μL^{-1} porcine kidney ACE (Sigma-Aldrich, Milan, Italy) solution in buffer 1 with 10 μM $ZnCl_2$, were added. Samples were incubated at 37 °C for 1 h, the reaction was then stopped with 0.1 M HCl (125 μL). The aqueous solution was extracted with ethyl acetate (2 × 600 μL); the solvent was evaporated, the residue was dissolved in 500 μL of buffer 1 and then analyzed by HPLC 1200 Series (Agilent Technologies, Santa Clara, US) in the following conditions: column, Lichrospher 100 C18 (4.6 × 250 mm, 5 μm; Grace, Italy); flow rate, 0.5 mL min−1; detector, λ 228 nm; mobile phase, water and MeCN, gradient elution from 5 to 60% MeCN in 10 min and 60% MeCN for 2 min, then back to 5% MeCN in 3 min; injection volume, 10 μL. The determination of ACE inhibitory activity was based on the comparison between the concentrations of HA in the presence or absence of the inhibitor (Inhibitor Blank). The phenomenon of autolysis of HHL to give HA was evaluated by a Reaction Blank (sample with the higher inhibitor concentration but without enzyme). The percentage of ACE inhibition was computed considering the area of the HA peak with the following formula (Eq. (2))

$$\% \, ACE \; inhibition = (A_{IB} – A_N) \; (A_{IB} – A_{RB}) \times 100$$

where $Area_{IB}$ is the area of HA in the Inhibitor Blank (IB) sample (with enzyme but without inhibitor), $Area_N$ is the area of HA in the samples containing different inhibitor amounts, and $Area_{RB}$ is the area of HA in the Reaction Blank (RB) sample (without enzyme and with inhibitor at the highest concentration). IC50 value is the concentration needed to observe a 50% inhibition of ACE activity.

S1.3. In Vitro Dipeptidil-Peptidase-IV (DPP-IV) Inhibition Assay

The DPP-IV Inhibitor Screening Assay Kit (Cayman Chemical Company, Ann Arbor, MI, USA) was used to assess the CH and BCH samples' ability to influence DPP-IV activity. Samples had final concentrations of 0.5, 1.0, 5.0 or 10.0 mg/mL. More specifically, 50 µL of samples (30 µL 1X assay buffer, 10 µL of sample or vehicle, and 10 µL DPP-IV enzyme) were combined with 50 µL of substrate solution (200 µM H-Gly-Pro-7-amino-4-methylcoumarin) and incubated for 30 minutes at 37 °C. The Synergy H1 microplate reader (ex./em. 350/450 nm) was then used to measure the fluorescence intensity.

S1.4. Cell Culture Conditions

Caco-2 cells from INSERM (Paris, France) and STC-1 from ATCC (HB-8065, ATCC from LGC Standards, Milan, Italy) were routinely subcultured in DMEM containing 25 mM glucose, 3.7 g/L NaHCO3, 4 mM stable L-glutamine, 1% non-essential amino acids, 100 U/L penicillin, and 100 µg/L streptomycin (complete medium), supplemented with 10% heat-inactivated FBS. They were maintained at 37 °C in a 5% CO_2 atmosphere.

S1.5. Cellular DPP-IV Inhibition Assay

A total of 3×10^4 Caco-2 cells were cultured in complete growth medium (all Gibco reagents, Fisher Scientific, Milan, Italy) at 37 °C under an atmosphere of 5% CO_2. Two days after seeded, the spent medium was discarded and Caco-2 cells were treated with samples at 10 mg/mL concentration and/or vehicle for 15 min at 37 °C. Subsequently, 40 µL of H-Gly-Pro-AMC (20.0 µM) was added to each well, and the fluorescence signal was acquired for 10 min.

S1.6. Enzyme-Linked Immunosorbent Assays (ELISAs) Assays for GLP-1 Quantification in Cell Culture Supernatants

The GLP-1 secretion of STC-1 was measured using a GLP-1 ELISA kit (Sigma Aldrich Sigma-Aldrich, St. Louis, MO, USA). Thus, 6×10^2 STC-1 cells per well were seeded into the 96-well plates. The cells were exposed to either vehicle (control) or CH and BCH (10 mg/mL) in growth media for 2 h. After the treatment, the supernatant was collected, centrifuged for five minutes at 500 × g at 4°C, and then incubated in 96-well microplates coated with GLP-1 monoclonal antibody for 24 h at 4°C. After incubation, the wells were washed four times and the detection conjugate was added for 2 h. After other four washing, the substrate solution was applied and left for 20 min. The plate was read using a Synergy H1 microplate reader (Biotek Instruments, Winooski, VT, USA) at an excitation/emission wavelength of 355 nm/460 nm after the reaction was stopped using a stop solution.

S1.7. Degree of Hydrolysis (DH) of the Hydrolysates

The DH was determined by the o-phthaldialdehyde (OPA) assay, according to Nielsen et al. with some modifications [2]. This assay is based on the formation of an adduct between the α-amino groups of peptides and the OPA reagent. The assay consisted of mixing 200 µL of OPA reagent with 5 µL of BCH and CH, respectively. After 2 minutes of

https://doi.org/10.3390/xxxxx

incubation at 25 °C, the absorbance was measured at 340 nm using the Synergy H1 fluorescent plate reader (Biotek, Bad Friedrichshall, Germany).

S1.8. High-Resolution Mass Spectrometry Analysis (nLC-HRMS)

All samples were solubilized in 1 mL of water and then subjected to the desalting procedure using 100 µL of sample. ZipTip C18 pipette tips (80 µg) were used, following the manufacturer's protocol. The eluate was dried using a SpeedVac. Samples were reconstituted in 30 µL of 0.1% formic acid (FA). All samples have been analyzed at UNITECH OMICs (University of Milano, Italy) using Dionex Ultimate 3000 nano-LC system (Sunnyvale CA, USA) connected to Orbitrap Fusion™ Tribrid™ Mass Spectrometer (Thermo Scientific, Bremen, Germany) equipped with nano electrospray ion source. Peptide mixtures were pre-concentrated onto an Acclaim PepMap 100 – 100µm x 2 cm C18 (Thermo Scientific) and separated on EASY Spray column ES900, 25 cm x 75 µm ID packed with Thermo Scientific Acclaim PepMap RSLC C18, 3 µm, 100 Å. The temperature was set to 35°C. the peptides were eluted using mobile phase A (0.1 % formic acid in water) and mobile phase B (0.1% formic acid in water/acetonitrile 20/80, v/v) at a flow rate of 0.300 µL/min. The run started under isocratic conditions at 96.0% A / 4.0% B with a flow rate of 0.30 µL/min. These conditions were maintained until 3.00 min. The gradient then increased linearly to 72.0% A / 28.0% B at 103.00 min, followed by a further increase to 60.0% A / 40.0% B at 113.0 min. At 114.0 min, the gradient was rapidly shifted to 5.0% A / 95.0% B and maintained until 117.00 min.

Initial conditions (96.0% A / 4.0% B) were restored at 120.0 minutes and held until 123.0 minutes. A second washing step was applied by increasing the gradient to 5.0% A / 95.0% B at 126.0 min, maintained until 129.00 minutes, followed by re-equilibration to 96.0% A / 4.0% B at 132.0 minutes and held until 135.0 minutes. A final high-organic wash was performed by shifting again to 5.0% A / 95.0% B at 138.0 min, maintained until 141.0 minutes, after which initial conditions (96.0% A / 4.0% B) were restored at 144.0 minutes and maintained until the end of the run at 150.0 minutes. Two blank samples were run between samples to prevent sample carryover. MS spectra were collected over an m/z range of 375 – 1500 Da at 120,000 of the resolutions (m/z 200), operating in the data-dependent mode, cycle time of 3 seconds between masters scans. HCD was performed with a collision energy set at 35 eV. Polarity was set as positive.

S1.9. Statistical Analysis

All data sets were analyzed using One-way Anova followed by Tukey's post hoc analysis, or Two-way ANOVA followed by Sidak's multiple comparison test (GraphPad Software 9, San Diego, CA, USA). Values were expressed as means ± standard deviation; p-values ≤ 0.05 were considered significant.

References

1. Boschin, G.; Scigliuolo, G.M.; Resta, D.; Arnoldi, A. ACE-Inhibitory Activity of Enzymatic Protein Hydrolysates from Lupin and Other Legumes. *Food Chem.* **2014**, *145*, 34–40, doi:10.1016/j.foodchem.2013.07.076.
2. Nielsen, P.M.; Petersen, D.; Dambmann, C. Improved Method for Determining Food Protein Degree of Hydrolysis. *J. Food Sci.* **2001**, *66*, 642–646, doi:10.1111/j.1365-2621.2001.tb04614.x.
